# Phydbac "Gene Function Predictor" : a gene annotation tool based on genomic context analysis

**DOI:** 10.1186/1471-2105-6-247

**Published:** 2005-10-12

**Authors:** François Enault, Karsten Suhre, Jean-Michel Claverie

**Affiliations:** 1 Structural and Genomic Information, CNRS – UPR 2589, 31 chemin Joseph Aiguier, 13009 Marseille, France

## Abstract

**Background:**

The large amount of completely sequenced genomes allows genomic context analysis to predict reliable functional associations between prokaryotic proteins. Major methods rely on the fact that genes encoding physically interacting partners or members of shared metabolic pathways tend to be proximate on the genome, to evolve in a correlated manner and to be fused as a single sequence in another organism.

**Results:**

The new "Gene Function Predictor", linked to the web server Phydbac proposes putative associations between *Escherichia coli *K-12 proteins derived from a combination of these methods. We show that associations made by this tool are more accurate than linkages found in the other established databases. Predicted assignments to GO categories, based on pre-existing functional annotations of associated proteins are also available. This new database currently holds 9,379 pairwise links at an expected success rate of at least 80%, the 6,466 functional predictions to GO terms derived from these links having a level of accuracy higher than 70%.

**Conclusion:**

The "Gene Function Predictor" is an automatic tool that aims to help biologists by providing them hypothetical functional predictions out of genomic context characteristics. The "Gene Function predictor" is available at .

## Background

Annotating proteins of unknown biological function is still a major bottleneck in the exploitation of genomic information. The main approaches are all based on the recognition of sequence similarity, from which functional homology is inferred with various levels of confidence. Methods such as BLAST, PSI-BLAST [1] or Pfam [2] are used to automatically generate functional annotations to a sizable fraction of the genes in newly sequenced genomes. However, from 20% to 50% of genes [3] are still annotated as being of unknown function, either because they have no statistically significant matches in current databases or because they only match uncharacterized protein sequences from other organisms. To provide putative functional assignments to those proteins, comparative genomic approaches are now reaching beyond the simple recognition of sequence similarity [4-6]. The reliability of these new methods, often referred to as genome context analysis, is now steadily improving, due to the almost exponential increase in the number of fully sequenced genomes. They allow the detection of functionally linked proteins, either physically interacting partners or members of shared metabolic pathways or cellular processes. The functional association of proteins may cause their encoding genes (i) to be part of a shared transcriptonal unit (Operon or Gene Cluster method), [7-9] or to exhibit a chromosomal proximity conserved in several genomes (Gene Neighbor method) [10,11], (ii) to have evolved in a correlated manner (Phylogenetic Profiles method) [12] or (iii) to have fused as a single gene in another organism (Rosetta Stone method) [13,14].

Here we introduce the new "Gene Function Predictor" of our web software Phydbac [15] based on the results given by a combination of these non-homology based methods. This database proposes putative associations between *Escherichia coli *K-12 proteins as well as functional GO term predictions derived from these associations. A blast mode is also available to apply the method to any protein sequence. In this study, we first describe separate improvements to the three major genomic context methods. An integrated score combining their results is defined and shown to predict protein pairwise associations more accurately than the ones already proposed in established databases such as Predictome [16], Prolinks [17] and String [18]. We then take advantage of the pre-existing functional annotations of the putatively associated proteins to assign them to GO categories [19]. The "Gene Function Predictor" proved to be particularly useful for the «conserved hypothetical protein» subset, as shown on a specific example.

## Implementation

This web tool is designed as a CGI script written in Perl running on an Apache web server. This script first retrieves genes through the process of the information entered into a HTML Form. A target gene can either be retrieved by its name or by the presence of a keyword in its annotation. The putative associations and functional predictions are then extracted by running a number of Perl scripts on a database of pre-computed blast hits and auxiliary information. Results for the query are then displayed through HTML pages. The "Gene Function Predictor" is accessible through any browser.

## Results and discussion

### Data sources and scoring

**Figure 1:**
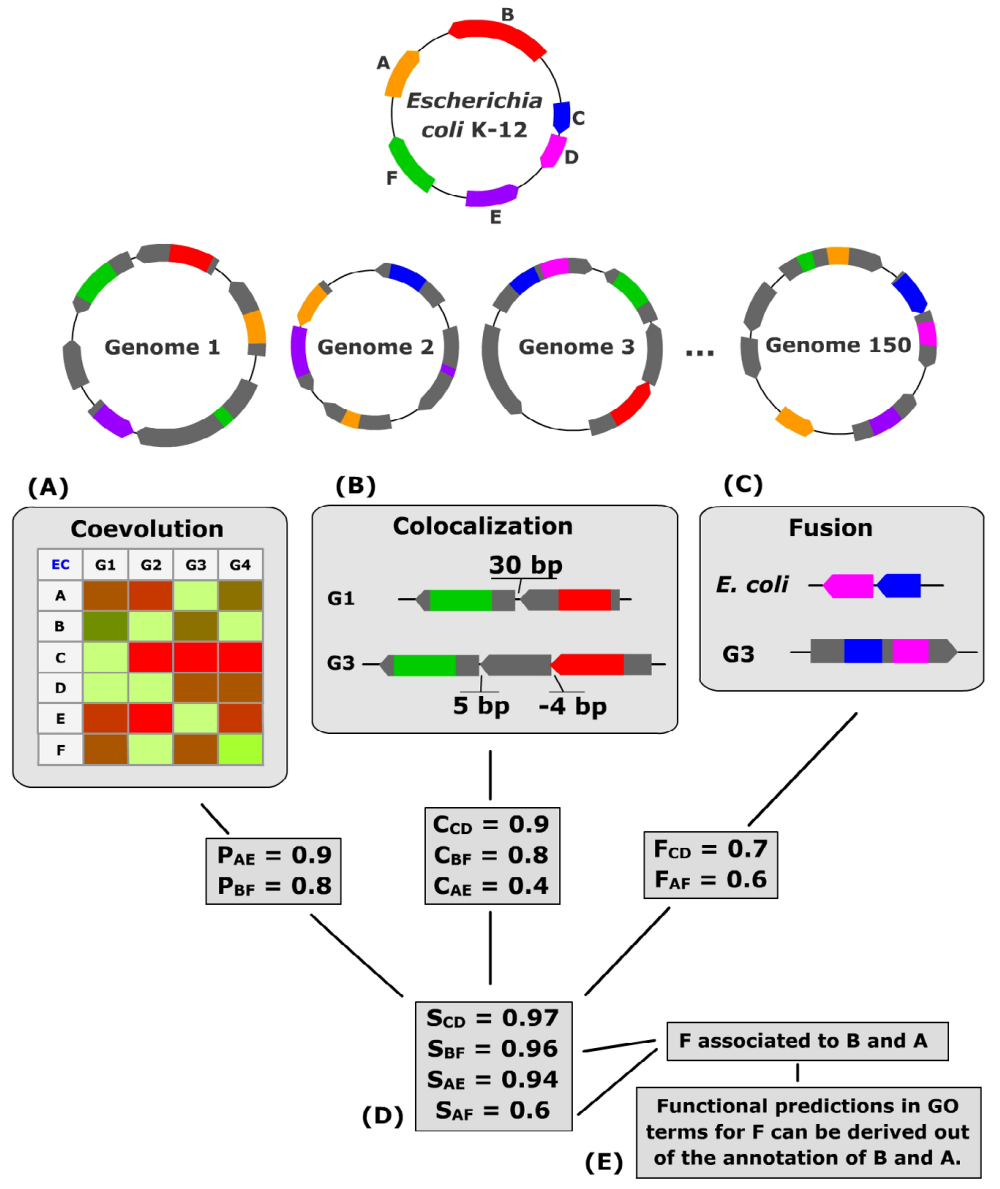
**Description of the methods used in the "Gene Function Predictor"**. The protein coding genes of our target organism *E. coli *are compared to the ORFs of 150 genomes. (A) The P score applied to *E. coli *protein phylogenetic profiles allows to identify protein pairs that evolved in a similar manner. For example, genes A and E are present in genomes 1, 2 and 150 and absent in genome 3. (B) The C score is associated to gene pairs nearby in, at least, one genome. This score is computed from the intergenic distances between *E. coli *genes and their respective homologs in all other genomes. The genes B and F (respectively red and green) are found only separated by 30 bp in genome 1 and by 5 bp in genome 3, resulting in a C score of 0.8 between those two genes. (C) The F score is computed for each domain fusion detected. In the example, domains of *E. coli *genes C and D are found fused in a gene of genome 3. (D) Significant P, C and F scores are combined in an integrated score. (E) Functional predictions are made out of the annotations of associated partners.

In this study, genomic context analysis is applied to the well annotated bacterium *Escherichia coli K-12 *(Figure 1). This analysis is performed using the 150 completely sequenced organisms available in Refseq, including 130 bacteria, 17 archaeal bacteria and 3 unicellular eukaryota. *E. coli *protein associations available in Phydbac's "Gene Function Predictor" are generated by three genomic methods : the phylogenetic profile, the colocalization and the Rosetta Stone methods. Improvements to these different methods and their implementation are described in the following.

### Consensus phylogenetic profiles (P)

It is established that proteins evolving in a correlated manner tend to participate in common metabolic pathways or constitute multi-molecular complexes. Using the simplest type of phylogenetic profile, the co-occurrence of genes is represented by a string of bits, each bit recording the presence or absence of an ortholog to a given gene in a genome [12]. In an earlier work [20], we proposed to replace this binary scale by continuous values derived from alignment scores.

Let *S*_*ab *_be the best Blastp bit score between a target protein *a *and all proteins of a bacteria *b *and *s*_*aa *_the self-score of the *a *protein aligned with itself. Each point of the phylogenetic profile of a protein *a *is computed as : *R*_*ab *_= *S*_*ab*_/*s*_*aa*_.

Each point of a profile is weighed proportionally to the length and quality of the corresponding alignment. Although this method was shown to improve on the binary method of Pellegrini et al. [12], the profiles are noisy. As we want no orthologs to be missed, even low sequence similarities are considered, bringing back a certain amount of false positives.

**Figure 2:**
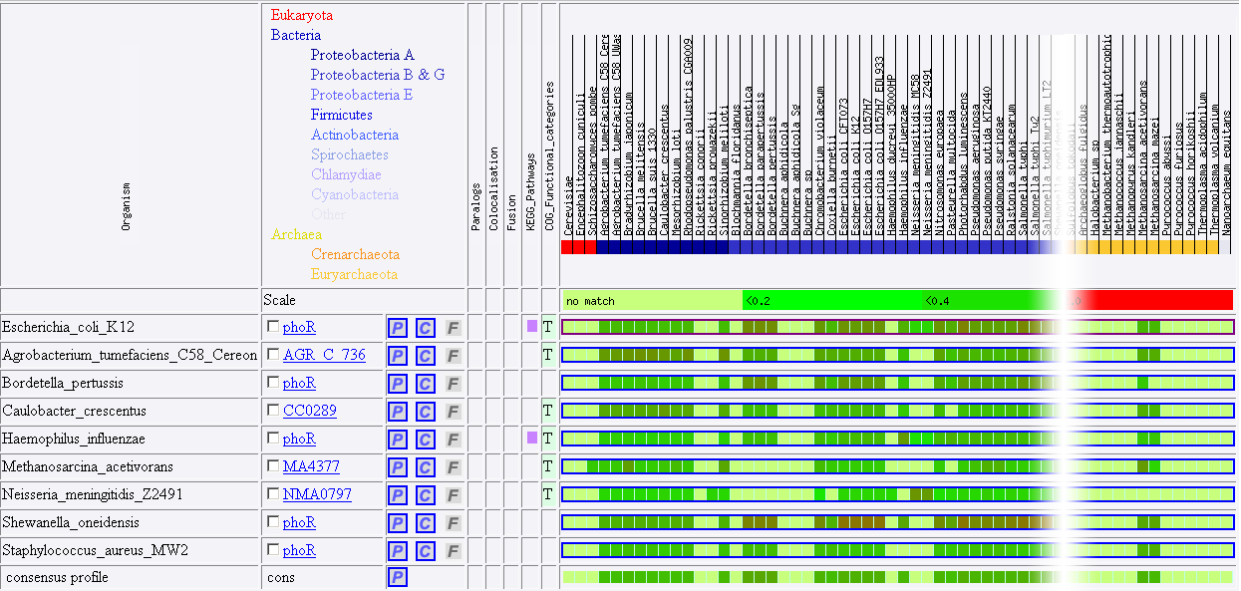
**Profiles of the *E. coli *protein phoR and of its best homologs in different organisms**. The consensus profile (CPP) of phoR is derived from these profiles as described in the text.

To improve their quality, profiles now use the information contained in the profiles of genes from other species. Introduced as a display feature in our web software Phydbac [15], Consensus Phylogenetic Profiles (CPP) are built from the profiles of a target gene and of its putative orthologs. The CPP of a gene has a non-zero score in a given column (corresponding to a bacterium) if more than half of its best matches in the other species has a match in this bacterium. The score of the profile for this column will then be the mean of the non-zero scores of the different putative orthologs with the corresponding bacteria. Figure 2 shows the profile of *E. coli *protein phoR, the ones of its best homologs in different organisms and the CPP of phoR built out of all those profiles. We note that the CPP of phoR is similar to its simple profile except at the columns corresponding to the two *Neisseria meningitidis *strains. Unlike phoR for which low sequence similarity matches are found in these strains, its best homologs do not exhibit any matches in these two organisms, suggesting that no orthologs of phoR are present in them.

From the CPP of the 4271 protein coding genes of *E. coli*, a pairwise P score is then computed. The P score is a correlation coefficient computed without the mean between each pair of profiles, profiles being N-dimensional vectors, with N the number of sequenced genomes (here N = 150). The profiles are stored in a matrix *R *where *R*_*ik *_is the value of the gene *i *profile at the column *k *corresponding to bacteria *k*. The score *P*_*ij *_reflecting the coevolution level between two genes *i *and *j *is then given by :



Pairs of gene products exhibiting the highest P scores are the most likely to be functionally linked.

### Detection of co-localizations (C)

The identification of pairs of genes part of the same operon in a genome can also lead to their functional associations. Indeed, genes organized into operons, i.e. genes transcribed into a single mRNA, are co-regulated and tend to fill related roles in cellular processes of the organism. Such genes are identified either by using intergenic distances separating genes [8], by analyzing conserved chromosomal adjacency between genes in a set of genomes [9-11] or more recently by combining these two sources of information [21]. Because the assumption that genes separated by small intergenic distances are likely to belong to a shared operon is true in all prokaryotic organisms [8,22], the intergenic distances in all the genomes are more informative than conserved chromosomal adjacencies. Our score C is based on intergenic distances separating colocalized gene pairs across all the genomes. Two genes are said to be colocalized in a genome if these two genes and the genes between them on the chromosome are on the same strand and if all adjacent gene pairs of the string are separated by less than 300 bp [11]. In *E. coli*, more than 98% of the gene pairs being on an operon described in RegulonDB [23] are separated by less than this threshold of 300 bp. The distance associated to a colocalized gene pair is the maximal intergenic distance found between them. For example, for three adjacent genes A, B and C on the same strand, if A and B are separated by 5 bp and B and C by 75 bp, we consider A and C to be colocalized with a distance of 75 bp.

To avoid artefacts due to the presence of redundant strains and of evolutionary close species in the 150 genomes, we restrict our analysis to 87 groups of similar organisms, made on the basis of the multiple alignments of the 150 homologs of three conserved genes [15]. A pair of genes found colocalized in *Xanthomonas campestris *and in the two *Xylella fastidiosa *strains will only be considered as colocalized in the group containing these organisms, the minimal distance separating this couple in a genome of this group being recorded.

The colocalization score C reflects the degree of confidence in the fact that genes are colocalized because of functional relationships, ie that genes are part of an operon in a genome. The score between each gene *i *and *j *of a target genome is :



where *Gij *are the groups of genomes where *i *and *j *are colocalized and *d*_*ij*_*(g) *the minimal distance in base pairs that separates *i *and *j *in a genome of the group *g*. The definition of C was derived from observed characteristics of colocalized genes. A colocalization score between two genes must always increase as groups in which these genes are co-localized are found. Our C score was built in order to verify this point. Indeed, C is equal to 1 minus a product of elements, each element, involving an intergenic distance, being comprised between 0.5 and 1. To calculate these elements, an exponential function is used because the information gained from an intergenic distance must not be proportional to the length of this distance. Different formulas were tested and this C definition is the one that gives the better results.

In contrast to the Operon or Gene Cluster method, our score C is able to detect gene pairs distant in *E. coli *that form an operon in other organisms (like genes B and F in Fig. 1). Unlike the Gene Neighbor method, it can detect operons present in only one organism (like genes A and E in Fig. 1). Of course, not all of the *E. coli *gene pairs are separated by less than 300 bp in at least one genome. Only 199,262 of the possible 9,118,580 gene couples of *E. coli *are found separated by less than 300 bp in at least one genome, with an average score C of 0.48. A C average of 0.87 is found when considering the 2219 pairs of genes present in the same operon described in RegulonDB [23].

### Identification of gene fusion events (F)

Associations of genes can also be deduced with the Rosetta stone technique [13,14] by detecting gene fusion events. Two distinct genes of a given organism that are found fused as a continuous sequence (referred to as the Rosetta Stone sequence) in another genome tend to physically interact. Non-homologous proteins fused as a single sequence are identified with the aid of the Pfam protein domain database [2]. Rpsblast of all the Pfam domains against all the proteins of 150 genomes and *E. coli *were computed, using a threshold of significance of 10e-10 for the expectation value of the alignments. Two *E. coli *proteins are determined to be fused if at least one domain of each protein is found separately in a third protein of another organism. As domains are relatively short compared to protein sequences, we did not consider overlaps larger than 10 residues between the alignments of the two domains on the Rosetta Stone sequence. The presence of two domains in different proteins as well as on the same coding sequence is of course not enough to be sure that a real fusion event took place between genes coding these proteins. But as such domains are likely to be functionally linked, it is also true for the proteins in which the domains appear separately. A score F is deduced from the probability that two genes are found fused by chance in another single sequence described in [17]. This score F depends on the number of sequences with which the two domains considered exhibit a significant sequence similarity and the number of sequences in which these domains are found fused. The score F is computed for each of the 22,100 *E.coli *protein pairs for which a putative domain fusion is detected.

### Evaluation and comparison between P, C and F and the integrated score

As the three scores P, C and F are based on different concepts, they are supposed to be independent and to provide different valuable information. To integrate them appropriately into a unique score, we have to scale them by their respective accuracy to predict genes that are functionally linked. As P, C and F are continuous scores, a list of ranked significant associations given by each approach allows to calculate the fraction of associations involving two genes annotated in the same COG category among associations linking COG-annotated genes [24].

**Figure 3:**
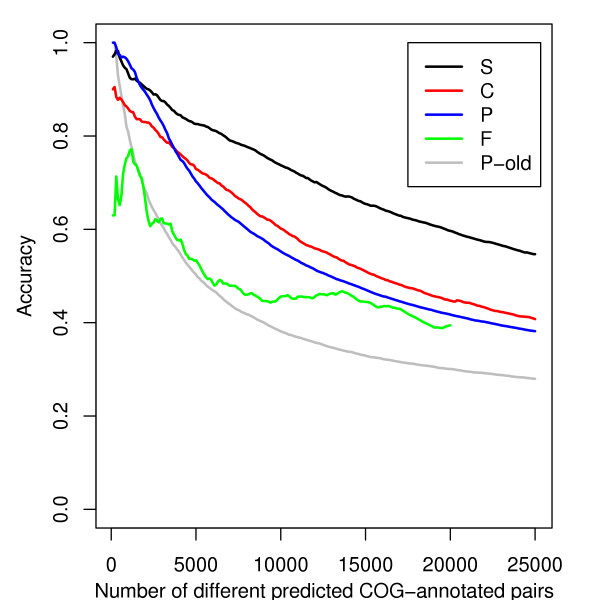
**Cumulated accuracy for the different methods**. The cumulated accuracy is the fraction of gene pairs associated by a method and being in the same COG category. The different curves represent this accuracy among the best associations for the P score based on Consensus Profiles, for the P-old based on simple profiles, for the score of colocalization C, for the F score detecting fusion events and for the integrated score S.

This success rate also allows us to compare the quality of each score (Figure 3). First of all, we note that the information on coevolution is better retrieved when Consensus Profiles are used (P) compared to our previous simple profiles (P old). The increase of accuracy between P and P-old is higher by more than 30% for any number of predicted pairs. C gives even better results than P, with 15,600 predictions with an accuracy higher than 0.5 (12,800 for P). 1,743 of the 2,219 gene pairs in shared operons of RegulonDB [23] have a C score higher than the threshold corresponding to an accuracy of 0.5. The score F associates 5,500 different *E. coli *protein pairs with a success rate higher than 50%.

The success rate is used to establish normalized scores across the different approaches. This normalization procedure then allows the individual scores to be merged into an integrated score in a simple way :

*S*_*ij *_= 1 - [(1-*P*_*ij*_) × (1 - *C*_*ij*_) × (1 - *F*_*ij*_)]

where *i *and *j *are two genes and with *P*_*ij*_, *C*_*ij *_and *F*_*ij *_set to 0 when no significant score is found for *i *and *j*. The quality of predictions made with this integrated score S is significantly better than each of P, C and F on their own (figure 3). For the 10,000 best associations between COG -annotated genes given by each method, the score S has a cumulated accuracy 21% better than the score C, 30 % better than the score P and more than 60% better than the score F alone. For an expected success rate of at least 80 %, 9,379 pairwise associations are derived from the S score, involving 2,500 *E. coli *genes. A coverage of 70 % (2,975 of the 4,278 genes) is obtained when considering an accuracy of 70 %.

### Comparative benchmarking of databases

There are three major databases of putative associations between prokaryotic genes derived from genomic context analysis : Predictome, String and Prolinks. Each one implements different methods with personal flavours. In Predictome [16], phylogenetic profiling, gene neighbor and domain fusion are implemented in their traditional way and applied to orthologous families of genes defined in COG. One of its major limitation is the absence of a quality score for each prediction. In the earlier releases of String [25], genomic analysis was also relying on COGs. A protein mode is now available, based on continuous phylogenetic profiling, gene neighbor, fusion, as well as experimental data and literature mining [18]. Prolinks [17] uses binary profiles not based on COG data. For pairwise alignments, the homology is considered significant if the e-value associated is lower than 10e-10. Text mining, gene fusion, gene neighbor as well as a gene cluster method are also implemented. For each method, they developed their own probabilistic score. Prolinks and String scale the different methods separately and then compute a confidence score.

**Figure 4:**
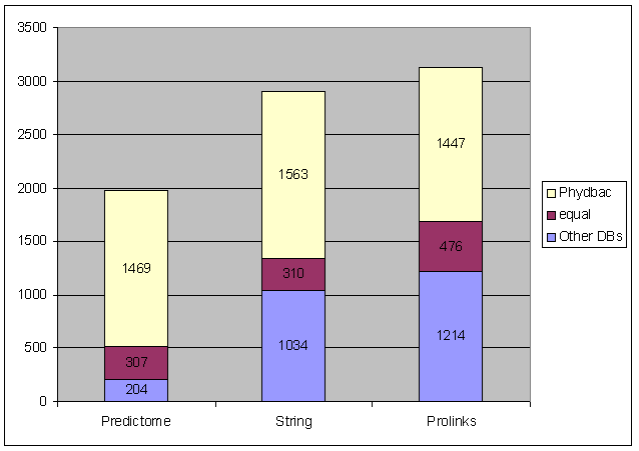
**Comparison of the databases**. Comparison of the results given by Phydbac and those found in the three existing databases based on non-homology methods.

To compare those three databases to Phydbac, the associations were downloaded from their respective web sites. As the accuracy of the putative links given by each database is tested against Gene Ontology data [19], we only keep associations involving GO-annotated genes. 18,760 different associations between GO-annotated genes are found in Predictome, 57,266 in String and 59,260 in Prolinks. For each database, each target gene annotated in at least a GO class has a certain number of GO-annotated genes associated to it. We select the same number of our best GO-annotated predictions involving this target gene and determine which database has the best accuracy for each gene (Figure 4). Associations given by our method more often imply genes belonging to the same GO category than associations of other databases.

We can note that Predictome gives better results for only 10 % of the genes (74% for Phydbac). This point is not surprising as the release of Predictome is the oldest of the three databases. String is the database that gives the most different results to ours (only 11% of genes having similar results). As we have seen, additional information, different to genomic information, has recently been added and the gene cluster method is not used. In figure 4, we note that results of Phydbac are more accurate than those of String for 54% of the 2,907 GO-annotated genes that have at least one association in String. Our putative associations are also better than those found in Prolinks. For 46% of the 3,137 GO-annotated genes of Prolinks, putative associations predicted by Phydbac imply two genes of the same GO category more often than those found in Prolinks. A surprising result described in the Prolinks paper (Bowers et al. 2004) is the fact that the integration of their 5 methods do not give better results than their Gene Neighbor method on its own. Their final score for a gene couple is the maximum value found with the 5 methods. As we have seen, the different methods are supposed to give independent information, and although this is not strictly true, a combination of the different scores (as in String and Phydbac) works better.

### Assignment to GO categories

In addition to putative associations, we developed an annotation procedure meant to assign genes to Gene Ontology categories [19]. GO provides structured classifications that cover several domains of molecular and cellular biology. Gene products are described throughout three non-overlapping domains : (i) Molecular Function describes activities at the molecular level, (ii) Biological Process describes biological goals accomplished by one or more molecular functions and (iii) Cellular Component describes locations at the level of subcellular structures and macromolecular complexes. GO can be viewed as a directed acyclic graph that represents a network in which each term may be a "child" of one or more "parents". For example, the function term "peptidyl-serine ADP-ribosylation", from the biological process vocabulary, is a child of both terms "protein amino acid ADP-ribosylation" and "peptidyl-serine modification".

In our annotation procedure, each term is considered as an independent class. For a target gene and for a fixed accuracy threshold for S, a certain number of genes are potentially linked to the target, associated to a total of *t *annotations. Each GO term *A *appears *n*_*A *_times in the *t *annotations (cases where *A *is a parent of one of the *t *annotations are also counted) and *N*_*A *_times in the total pool of the *T *annotations of *E. coli *genes. The probability to draw at least *n*_*A *_annotations of the GO term *A *or of child terms of *A *by chance out of *t *annotation is given by :



For each target gene, GO terms with a value for this probability lower than 10e-10 are considered as putative functional annotations. The same procedure is repeated for decreasing accuracy thresholds of S.

Considering *E. coli *genes already annotated with GO terms, 1,725 GO term predictions are derived from the 4,006 links with expected success rate of 90%. 80% of these 1,725 predictions are correct, i.e. already appear in the annotations of genes. Out of these 1,725 predictions, the 974 best, corresponding to a probability lower than 10e–13, have an accuracy greater than 85 %. When using links with an expected success rate of 80% (9,379 pairwise links), 70% of the 6,466 functional predictions are correctly inferred. Of course, predicted GO terms that do not appear in the gene's annotation cannot be considered systematically as false predictions. Annotated genes may have additional – yet unknown – functions or predictions may represent the gene function on another level. For example, yaeT, annotated in GO term as an "outer membrane"protein, which describes its location, is predicted to participate in lipid A biosynthesis and metabolism, which describes the biological process it may be involved in. For an accuracy threshold of 60%, 16,280 GO term predictions are made for more than 1,500 *E. coli *genes.

### Web interface and example

The "Gene Function Predictor" emulates two main different modes of operation. In the first mode, the predictions can be made for any protein sequence pasted by the user in a similar manner to Plex [26]. In this Blast mode, the consensus profile of the given sequence is dynamically created and the most similar profiles are determined among the genes of the organisms processed in Phydbac. The conserved neighbors on the chromosomes are also determined by comparing the sequences found proximate to the pasted sequence in all organisms. Genes associated to the query by Rosetta Stone are identified by the presence of conserved domains in this sequence. If some associated partners are determined, an annotation procedure similar to the one described above is applied, even though all the partners do not come from the same organism. This mode of operation is useful for genes of organisms whose sequence is not complete or not public.

**Figure 5:**
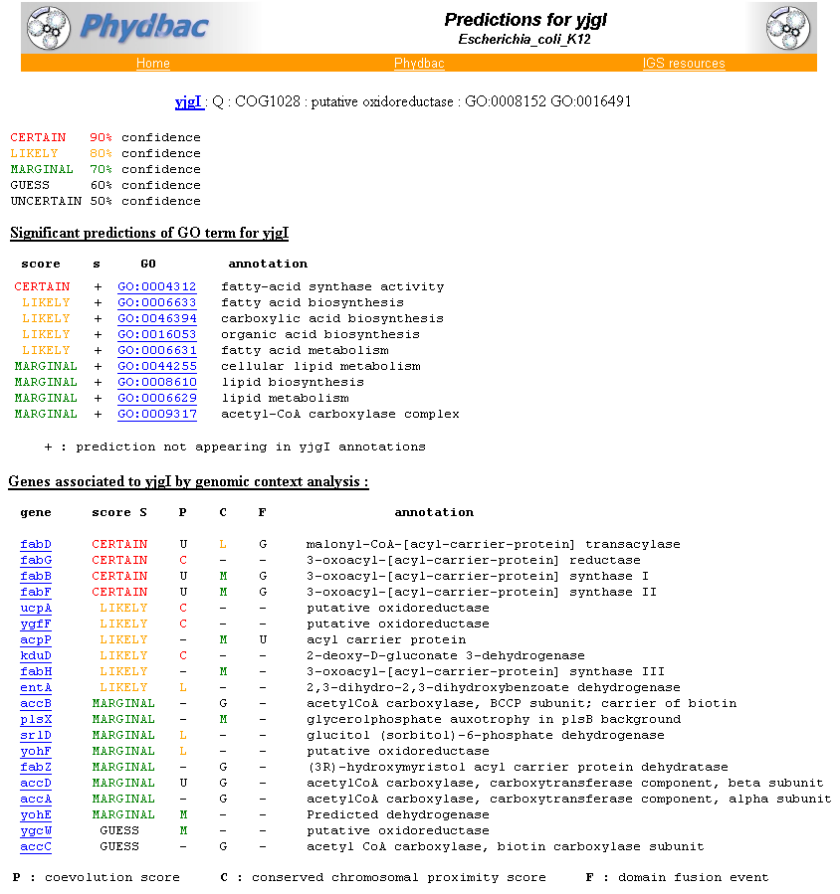
**Typical output of the « Gene Function Predictor »**. Predictions for the *E. coli *gene yjgI. Significant predicted GO terms are displayed as well as the associations from which these predictions are derived.

The second mode of operation of the "Gene Function Predictor" is a database gathering the results described in the study for processed organisms. Currently limited to *E. coli*, this mode will be extended to all fully sequenced micro-organisms. *E. coli g*enes can be retrieved by their names or by the presence of a keyword in their annotations. For any gene queried, its most probable association partners as well as its significant GO term predictions are displayed on a single page. The confidence we have in the different predictions is depicted through keywords and colours. For example (Figure 5), yjgI, a protein annotated as "putative oxidoreductase"is associated to a reductase (fabG) and to other putative oxidoreductase (ucpA, ygfF) by coevolution (P) and 4 of its 7 best associated partners are acyl-carrier proteins and are significantely linked with yjgI by each of the three methods (P, C and F). As acyl carrier proteins are fundamental components of fatty acid biosynthesis, the best GO term predicted for yjgI is "fatty-acid synthase activity"and "fatty-acid biosynthesis"(Figure 5). The specific biochemical activity of yjgI cannot be deduced from these results, but like its most probable partners, yjgI is likely to be involved in fatty acid synthesis. Furthermore, acyl carrier protein as well as acyl carrier protein synthase are known to be essential for *E. coli *viability. Maybe this is also the case for yjgI. For such proteins annotated as "putative ..."or uncharacterized proteins, our tool provides hypothetical functions, either new or on another level of description. The "Function Predictor" is fully linked with the software Phydbac, as a closer analysis and additional information can be retrieved through the display of the profiles, of the conserved gene neighbors and of the gene fusion.

## Conclusion

Although the huge amount of data provided in the past few years from genome sequencing allows a large spectrum of research axes, the most serious problem of modern bioinformatics is still the quality and degree of completeness of the annotation of sequenced genomes [3]. Earlier versions of Phydbac made a step in this direction by providing an interactive resource on prokaryotic proteins and on their context that may help microbiologists. But the different sources of information contained in genomic data may not always be trivially extracted by hand. The new "Gene Function Predictor"integrates the different concepts to automatically predict putative functions for *E. coli *genes.

We have shown that the integrated score, from which the putative pairwise associations are derived, gives better results than any intermediate approach on its own. We also compared our results to the best associations found in major databases based on the same concepts. Our protein linkages proved to be more accurate.

GO assignments were also benchmarked and are highlighted with distinct colors when displayed on the web. As GO is an annotation standard, the same procedure can be computed for any prokaryotic organism. A future version of the "Gene Function predictor", currently limited to *E. coli*, will be extended to all fully sequenced micro-organisms, even though a Blast mode of operation is already available.

## Availability and requirements

Project name: Phydbac "Gene Function Predictor"

Project home page: 

Operating system(s): Web server

Programming language: Perl and HTML

## Authors' contributions

FE and KS analyzed the data and designed the methodology. FE developed the programs, the web interface and wrote the manuscript. KS contributed with ideas on overall design, feature requirements, and implementation. JMC coordinated the research and JMC and KS assisted with drafting the manuscript.
